# An Evaluation of e-Health Service Performance through the Integration of 5G IoT, Fog, and Cloud Computing

**DOI:** 10.3390/s23115006

**Published:** 2023-05-23

**Authors:** Salman A. AlQahtani

**Affiliations:** New Emerging Technologies and 5G Network and Beyond Research Chair, Department of Computer Engineering, College of Computer and Information Sciences, King Saud University, P.O. Box 51178, Riyadh 11543, Saudi Arabia; salmanq@ksu.edu.sa

**Keywords:** cloud computing, e-Health services, fog computing, Internet of Things, queueing networks

## Abstract

In recent years, Internet of Things (IoT) advancements have led to the development of vastly improved remote healthcare services. Scalability, high bandwidth, low latency, and low power consumption are all essential features of the applications that make these services possible. An upcoming healthcare system and wireless sensor network that can fulfil these needs is based on fifth-generation network slicing. For better resource management, organizations can implement network slicing, which partitions the physical network into distinct logical slices according to quality of service (QoS) needs. Based on the findings of this research, an IoT–fog–cloud architecture is proposed for use in e-Health services. The framework is made up of three different but interconnected systems: a cloud radio access network, a fog computing system, and a cloud computing system. A queuing network serves as a model for the proposed system. The model’s constituent parts are then subjected to analysis. To assess the system’s performance, we run a numerical example simulation using Java modelling tools and then analyze the results to identify the key performance parameters. The analytical formulas that were derived ensure the precision of the results. Finally, the results show that the proposed model improves eHealth services’ quality of service in an efficient way by selecting the right slice compared to the traditional systems.

## 1. Introduction

The Internet of Things (IoT) and eHealth services have led to a powerful transformation of the traditional medical community. The IoT refers to the ability of a physical object to connect, send, and receive data [1]. Integration with cloud computing has led to a wide range of benefits and applications, enhancing the limited processing capabilities and storage of IoT devices. In the healthcare context, the IoT–cloud system can be used to provide and deploy eHealth services for remote diagnosis and treatment “at any time”, where cloud computing can help in storing, analyzing, and virtualizing the collected real-time patient data [2,3]. This will minimize hospitalizations, healthcare costs, and pressure on health professionals [4]. Furthermore, transferring real-time sensor data to cloud services assists in monitoring and predicting patient emergencies before they occur, which results in the improvement of the healthcare quality [5]. However, many eHealth services are critical and time-sensitive, and the delay between producing the data at IoT sensors and making a reaction in cloud computing data centers (CCDC) is not tolerable. As an example, we cannot accept a delay in the alarm systems for emergency situations. To overcome this issue, fog computing close to the IoT devices is inserted to reduce the distance between the CCDC and the medical sensors [6]. The impact of adding fog nodes on the network edge is significant for sensitive eHealth applications because it provides real-time processing, low latency, and fast reaction time [2,7]. Moreover, the decentralized fog/edge analytics reduces the load on the network and cloud servers, thereby enhancing the security and privacy for health data [8]. Fog nodes can be accessed from the edge network, unlike the cloud servers that need the core network [6]. By using the IoT/fog/cloud model, the data can be preprocessed and aggregated at the fog nodes, and only those data requiring greater capabilities move to the CCDC. However, Cisco predicted that 500 billion devices will be connected to the Internet by 2030 [9]. The growth of healthcare IoT (H-IoT) devices and the exponential growth in communications and its applications require the provision of different QoS requirements the emerging technology known as network slicing can meet. This enables the network elements and resources to be used in parallel while being isolated from each other, enabling the customization of services for H-IoT [5,10]. The main emerging technologies that enable network slicing are the software-defined network (SDN) and the network function virtualization (NFV). The SDN is useful for network slicing due to the separation between the network controls and the forwarding data, while the NFV aims to separate the functions from standalone boxes [5]. Accordingly, fog computing, SDN, and NFV technologies, combined with Cloud-RAN, are used to reduce the latency of eHealth services. Furthermore, fog nodes can use Cloud-RAN information, and Cloud-RAN can use fog services in return [11]. Cloud-RAN enables network slicing because it separates base station functions into distributed and centralized units. Centralization means that the baseband resources are grouped in a remote pool known as the baseband unit pool. The virtualized baseband unit (virtual BBU) pools provide factuality measures by reducing the time it takes to send and receive data and reducing the network cost and scalability. The remote radio heads (RRHs) distributed in the region require communication with and connection to the BBU via high-data rate single-mode fibers. An RRH contains an amplifier, multiplexing, and a transformer for converting data from digital to analog, and vice versa, thereby reducing the time spent sending and receiving data [12,13].

Healthcare IoTs require profitable network features. The major features of smart healthcare services are low latency, high bandwidth, high reliability, and a high battery lifetime [10]. The major trends in e-Health services are *online consultation*, *health monitoring*, and *remote surgery* [14]. Each service has specific requirements to achieve the desired system performance. In online consultations, the patient monitors the biological sensor’s data at home and sends it to an expert or hospital for a real-time video-based consultation. Online health monitoring makes the hospital virtual, which means that attaching medical sensors to patients is a vital process in human real-time streaming, analysis, and monitoring. In this type of system, healthcare providers at a hospital can check the registered patients and monitor their status. In a remote surgery system, tactile application is required due to system sensitivity and the need for high reliability and low latency when receiving and sending back data. In this type of system, the robotic control data are transferred in one direction, and the streaming images and biological data are transferred in the other direction. However, any data loss can be significant because human life could depend on it [15,16,17]. Example of medical sensors include temperature probes, force sensors, heart rate sensor, and pressure sensors. Table 1 summarizes the major QoS requirements for each service type.

For the eHealth system in this study, we propose an architecture composed of H-IoT sensors, fog computing, and CCDC. The proposed model can improve eHealth services’ quality-of-service demands in an efficient way compared to the traditional systems. The system is divided into three logical systems using fifth-generation (5G) network slicing technology. Each system is used for a certain class of healthcare services according to QoS requirements. Fog computing nodes are used for sensitive eHealth applications that require low latency and real-time processing. We model each subsystem using a queueing network to estimate the main performance metrics. The main focus of this study is to improve the performance of eHealth systems and the overall QoS requirements.

Based on our knowledge, the proposed model herein is different from the published models in at least one of the following senses:

1. An architecture composed of IoT/fog/cloud is provided for the main e-Health services;
2. The e-Health slices herein use three sequential subsystems (i.e., Cloud-RAN, fog, and CCDC) are modeled; iii): each subsystem is derived using the M/M/i/J queueing theory with a finite capacity J; and
3. The main motivation is to enhance the QoS requirements for healthcare systems.

The main contributions of this paper are as follows:

1. designs an analytical model based on the queueing theory to calculate and estimate the main performance parameters of eHealth services (e.g., system throughput and system drop rate);
2. provides three classes of eHealth services, each presented with a queuing model composed of concatenated subsystems (Cloud-RAN, fog, and CDC);
3. establishes the key performance metrics of the network: the mathematical formulas for the analytical model are presented and discussed in a dedicated section for each of the three subsystems;
4. provides an example, including numerical data to demonstrate the work, calculates the main performance measures, and estimates the number of computing resources required in each subsystem; and
5. uses a Java modeling tool (JMT) simulation module to confirm the correctness of the proposed paradigm.

The rest of the paper is organized as follows: Section 2 presents the related works; Section 3 introduces the proposed eHealth system; Section 4 and Section 5 demonstrate the proposed model and the analytical performance metrics of each subsystem; Section 6 presents the simulation and results; and Section 6 provides the conclusion.

## 2. Related Works

Several studies that investigated the H-IoT and healthcare systems are discussed in this section. The authors in [18] presented four different scenarios or use cases for 5G network slices: enhanced mobile broadband (eMBB); critical communications; vehicular-to-everything (eV2X); and massive IoT. Several works introduced different types of slices, each with specified QoS requirements. The authors in [19] introduced a home-based elderly care system that consisted of two network slices: the healthcare network slice between patients and caregivers (eMBB) and the smart home network slice that aimed to improve security and efficiency for elderly people. This system generated automated alerts in case of accidents or other emergency situations. In [15], the authors proposed a privacy-preserving slice selection mechanism to increase efficiency and security for IoT devices. For secure access, session keys are shared between users, the local fog, and IoT servers. Service-oriented anonymous authentication and session keys are required to ensure user anonymity, authenticity and service data confidentiality. Meanwhile, the authors in [20] proposed a 5G-smart diabetes system that included three layers, namely a sensing layer that collected the patient data, a personalized diagnosis layer that analyzed the data using machine learning algorithms, and the data-sharing layer where the users’ social and data space were used to enable treatment supervision by relatives and friends. The authors in [21] presented a general framework for IoT applications that consisted of the IoT–fog–cloud architecture. To minimize the service delay for IoT nodes, they presented a collaboration and an offloading policy for fog nodes. In the proposed policy, the fog nodes either processed the request or offloaded it to the other neighbor fog nodes with fewer requests or to the cloud servers. Moreover, the authors in [22] showed another similar work that aimed to reduce energy consumption for a mobile getaway and reduce the service delay for healthcare systems. In the proposed architecture, a 5G-based smartphone was used as a getaway where decisions were made to process requests, either on the local device or when they offloaded to mobile edge computing. Another work worth mentioning is the one presented in [23]. This paper presented a queueing architecture based on cloud–fog computing for IoT nodes. The main focus of their work was how to handle increased workload on the middleware layer (fog and cloud nodes) by providing a dynamic scaling algorithm. The algorithm computed and monitored the value of the key performance parameters at every “T” interval. An additional number of fog nodes was deployed if the value exceeded a predefined threshold. The authors in [24] proposed the fingerprint mechanism for healthcare applications. This technology was used to customize resources and meet the requirements of healthcare applications to increase reliability. The authors in [8] studied the effect of fog computing because it provided low latency, real-time proceeding, and high response time requirements. Consequently, the performance and the QoS requirements of healthcare applications were improved. Meanwhile, the authors in [25] addressed the privacy issue in healthcare IoT applications. Their model used a mutual privacy-preserving k-means strategy where the cloud was included as a third party to reduce communication complexity.

## 3. System Architecture and Assumptions

**Figure 1:**
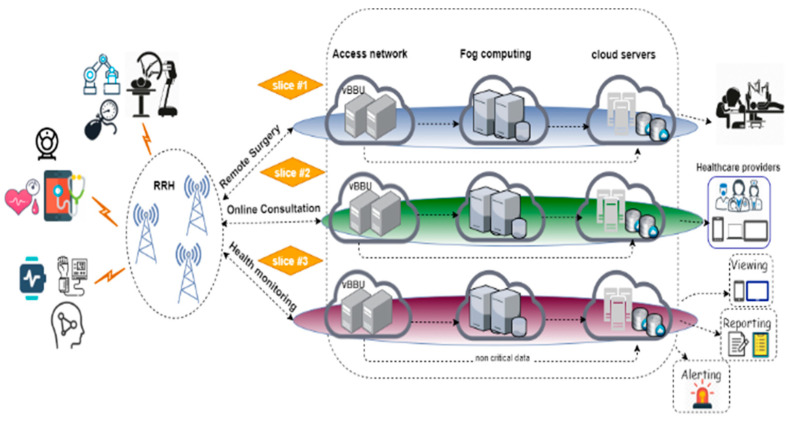
Architectural model of the H-IoT system.

**Figure 2:**
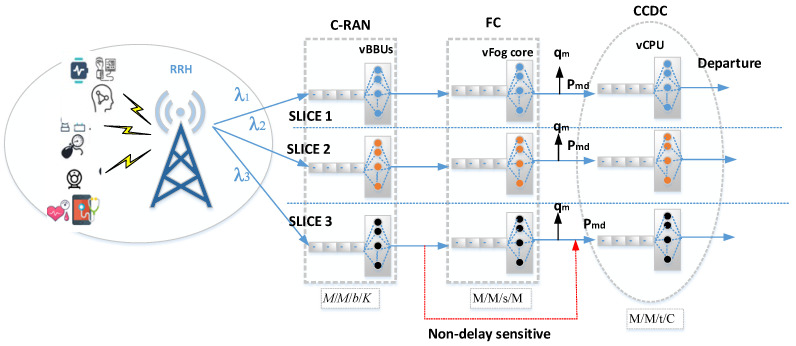
Proposed queueing model of the H-IoT system.

The proposed eHealth system consisted of three sequential phases for each 5G network slice. This system could divide the network into three slices according to the importance of collecting data from sensors and how fast it needed to be processed. Each slice contained Cloud-RAN, fog, and CDC, as shown in Figure 1. Using SDN and NFV technologies, the slices remained independent of each other because of the inherent isolating nature of the 5G slices. The data collected from the sensors were distributed between three slices: *remote surgery, online health monitoring, and online consultation*. The RRHs were responsible for sending to and receiving data from the IoT sensors and were assumed to be uniformly distributed over a certain range. The virtual BBU pool contacted with the RRH units through high-bandwidth links, such as a fiber. In the architecture illustrated in Figure 2, the RRH nodes sent the sensor data to the appropriate slice or virtual BBU pool according to the predetermined requirements. Each slice preserved several virtual BBUs based on the data processed. The incoming data from each end user arrived with an arrival rate of λ based on the Poisson process, where the total arrival rate is calculated according to the formula $\lambda =\sum_{i=1}^{m}\;\lambda_{i}$, in which “*i*” is an index for the total patients m. The service discipline is first in first out (FIFO), with no resource reservation. The system has an exponential distribution service time with rate mean μ.

The critical and sensitive data that required specific services, such as low latency and high bandwidth, were transmitted to the fog servers. In network fog computing, multiple services were provided, and there were multiple servers for this. The fog nodes generated alarms in emergencies if the data exceeded a given threshold value, for example, or transmitted the data to CDC servers for further processing and storage. The number of servers in the fog in each slice was “c”. The patient information was stored in the fog servers. This information was accessible by authorized doctors or caregivers to increase the accuracy of diagnosis and provide real-time information about patients.

The CDC was a set of servers that provided different services and included a database, network equipment, power distribution, and a cooling system. Many virtual and real servers were provided according to the corresponding available services. Large companies, such as Yahoo, Google, and Amazon, have physical servers hosted in enormous CDCs. In these CDCs, each server supports up to “T” virtual machines.

## 4. System Model

The use of a single 5G cell with one RRH and many eHealth sensors is considered. The eHealth system is served using 5G slices, where each service category is served using the required slice. Each eHealth slice includes three sequential queues, as shown in Figure 2. The C-RAN queue is defined as multiple cores with virtual BBUs. It follows the *M*/*M*/*b*/*K* model, where the first *M* indicates that the arrival process is Markovian and considers a Poisson process, and the second *M* defines the service process time that obeys the exponential service time. The number of virtual BBU servers in each C-RAN is equal to *b*. The number of users accepted in the system is finite up to *K*, including those in service. The sensors attached to the patients send the data packet to the C-RAN queue according to the predefined QoS requirements of the network slice. The data packet processing works on a FIFO basis. The arrival of a message from the sensors follows the Poisson process, with an arrival rate of $\lambda_{c}$. The processing time required for each data packet is exponentially distributed; hence, the expected time of service for each request is $1/u_{c}$. After the data packet is processed, it is transmitted to the fog subsystem if it is sensitive and critical, or sent directly to the CDC.

When the data packet is sensitive and critical, it will be processed in the fog by the fog network queue’s additional services. Each fog queue follows and “M/M/s/M” queuing model. The arrival rate for the packets arriving from the C-RAN is λ_m following the Poisson process. The service time is exponentially distributed, and the mean service time is equal to 1/μ_m. Let the number of virtual servers in each fog core be s. The capacity of each fog core is finite and serves up to “M” arrived packets. The packets are sent to the CCDC with probability (p) and leave the system with the probability of q = (1 − p) after they have been fully processed.

The final destination of the data packet is the CCDC network queue. This queue has an enormous number of computing data centers that include multiple cores. The virtual CPUs lie on the physical CPU cores. Data storage and processing are performed in the CPU cores. The CCDC network follows the M/M/d/C queueing method. The Poisson processing follows, with an arrival rate of λ_d. The service time is exponentially distributed, with an expected mean of 1/μ_d. The number of vCPUs in each core is ‘d’, and each core’s capacity is up to ‘C’ arrival packets. This system is illustrated in the following steps:

Initially, the H-IoT sensor attached to a body sensor network sends data packets to the distributed RRH. Later, the RRH sends packets to the virtual BBUs in a C-RAN subsystem. Further, the packet is put in a queue that meets the predefined requirements (slice). In each slice, the packet is processed by virtual BBUs and sent to either the fog network or CCDC network queue. In the fog network queue, the packet is processed and temporarily stored and then sent to the CCDC, or otherwise leaves the system if it does not need any further processing. Finally, the packet arrives in the CCDC queue to acquire more processing and storage.

## 5. Performance Analysis

In this section, each subsystem is analyzed, as is the overall system’s. The main performance metric formulas are obtained for the proposed queueing model. This section includes the main performance measurement vectors, such as throughput, utilization of CPU, the average number of packet requests, average number of packets waiting in the queue, average response time, the average waiting time, and rate of system loss. Finally, we compute the overall system cost in terms of the system cost and waiting time. In the proposed queueing network, the eHealth services are divided into three classes, and each has three subsystems.

### 5.1. Ran Subsystem

In this section, each virtual BBU core follows the M/M/b/K queueing model. Table 2 clarifies the parameters used herein. The M/M/b/K queueing model has multicores in each virtual BBU, and each virtual BBU core contains up to “K” packets in the subsystem. The arrival and departure of the data packet follow the birth–death process according to which the steady state of the system follows the continuous Markov chain process.

Following the Little theorem and the well-known M/M/b/K analytical model, the steady state of the C-RAN subsystem is expressed as follows:

(1) $$P_{k}=\left\{\begin{array}{l} \frac{1}{K!}\left(\frac{{\lambda_{c}}^{K}}{\mu_{c}^{K}}\right)P_{0},\;\;\;for\;1\leq K<b\\ \frac{\left({\lambda_{c}}^{K}\right)}{b!b^{k-d}\mu_{c}^{K}}P_{0},\;\;\;for\;K\geq b \end{array}\right.$$

the, $\rho_{c}$ is used to indicate the utilization of a single server and can be defined as:

(2) $$\rho_{c}=\frac{\lambda_{c}}{\mu_{c}}$$

The utilization of *b* servers in the system is expressed as:

(3) $$\rho_{b}=\frac{\lambda_{c}}{b\mu_{c}}$$

Let $P_{0}$ indicate the probability that when the system is empty, the $P_{0}$ is given by the normalization

(4) $$P_{0}={\left[1+\sum_{i=1}^{b-1}\;\frac{{\left(b\rho \right)}^{i}}{i!}+\frac{{\left(b\rho \right)}^{d}\left(1-\rho^{K+1-b}\right)}{b!\left(1-\rho \right)}\right]}^{-1}$$

The limitations of the packet volume accepted in the virtual BBU core give rise to blocking packets. A packet requests to join the system but is denied as the system is full. The probability of blocking packets is expressed as $P_{B}^{i}$.

(5) $$P_{B}^{i}=P_{K}^{i}$$

The average number of blocked packets is

(6) $$\lambda_{c}P_{K}^{i}$$

From this equation, the rate of effective packets is derived as follows:

(7) $$\lambda_{e}=\lambda_{c}\left(1-P_{K}^{i}\right)$$

The average throughput of the system is the total amount of packets served during a defined period of time expressed as

(8) $${{\bar{T}}_{c}}^{i}=\lambda_{c}\left(1-P_{B}^{i}\right)$$

The utilization of each virtual BBU core is expressed as

(9) $${\upsilon^{i}}_{c}=\frac{{{\bar{T}}_{c}}^{i}}{b\mu_{c}}=\frac{\rho_{b}}{b}\left(1-P_{B}^{i}\right)$$

The mean number of packet requests to join a single virtual BBU core is computed as

(10) $${{\bar{M}}_{c}}^{i}=\sum_{a=1}^{k}\;aP_{a}^{i}$$

The mean number of packet requests waiting in each virtual BBU core queue is obtained as shown:

(11) $${{\bar{Q}}_{c}}^{i}=\sum_{a=d+1}^{k}\;(a-d)P_{a}^{i}$$

Finally, the mean response time and the mean waiting time are obtained as follows using Little’s formula:

(12) $$\bar{E{{\left[R\right]}_{c}}^{i}}=\frac{{{\bar{M}}_{c}}^{i}}{\lambda_{c}\left(1-P_{B}^{i}\right)}$$

(13) $${\bar{E\left[w\right]}}_{c}^{i}=\frac{{{\bar{Q}}_{c}}^{i}}{\lambda_{c}\left(1-P_{B}^{i}\right)}$$

The performance metrics related to the Cloud-RAN subsystem are computed by deriving the main equations of the queueing network. First, the probability of denied (blocked) packets due to the shortage of space in all virtual BBU cores in Cloud-RAN is presented as

(14) $$P_{K}=\sum_{i=1}^{F}\;P_{K}^{i}$$

The mean number of packet requests in the Cloud-RAN subsystem is defined as follows:

(15) $${\bar{M}}_{c}=\sum_{i=1}^{F}\;{{\bar{M}}_{c}}^{i}$$

The mean number of packets arriving in the Cloud-RAN subsystem and waiting in the queue is expressed as shown:

(16) $${\bar{Q}}_{c}=\sum_{i=1}^{F}\;{{\bar{Q}}_{c}}^{i}$$

The mean throughput of the Cloud-RAN subsystem is expressed as follows:

(17) $${\bar{T}}_{c}=\sum_{i=1}^{F}\;{{\bar{T}}_{c}}^{i}$$

Finally, the mean response time and the mean waiting time for the Cloud-RAN subsystem are obtained:

(18) $$\bar{E{\left[R\right]}_{c}}=\frac{{\bar{M}}_{c}}{{\bar{T}}_{c}}$$

(19) $$\bar{E{\left[w\right]}_{c}}=\frac{{\bar{Q}}_{c}}{{\bar{T}}_{c}}$$

### 5.2. Fog Computing Subsystem

In the proposed queue model, the storage and computational resources are in the fog. The fog subsystem contains multiple fog cores. Each single core is modeled as an M/M/s/M queueing system. Table 3 lists each parameter required in this section.

The M/M/s/M queueing system contains a number of cores to process the incoming packets from the C-RAN and the maximum number of packets each core can process up to “M.” The system follows the birth–death process; hence, the steady-state probability of the system for each fog corresponds to the stationary probability of the Markov chain process.

The steady state is obtained as follows:

$P_{m}$ = probability m packets in the system.

(20) $$P_{m}=\left\{\begin{array}{l} \frac{1}{M!}{\left(\frac{\lambda_{m}}{\mu_{m}}\right)}^{m}P_{0},\;\;\;for\;1\leq m<s\\ \frac{1}{s!s^{M-s}}(\frac{\lambda_{m}}{\mu_{m}}{)}^{M}P_{0},\;\;\;for\;m\geq s \end{array}\right.$$

where, *ρ* indicates the utilization of a single server.

(21) $$\rho_{m}=\frac{\lambda_{m}}{\mu_{m}}$$

The utilization of *s* servers in the system is presented as follows:

(22) $$\rho_{s}=\frac{\lambda_{m}}{s\mu_{m}}$$

$P_{o}$ indicates the probability when the system is empty.

We obtained $P_{o}$ by normalization:

(23) $$P_{0}={\left[1+\sum_{i=1}^{s-1}\;\frac{{\left(s\rho \right)}^{i}}{i!}+\frac{{\left(s\rho \right)}^{s}\left(1-\rho^{M+1-s}\right)}{s!\left(1-\rho \right)}\right]}^{-1}$$

The blocking probability is the probability of denied requests of packet arriving at the fog core when the total number of packets in a single fog core is *M*. We obtain the blocking probability as follows:

(24) $$P_{B}^{i}=P_{M}^{i}$$

Here, $P_{M}^{i}$ represents the probability of packets that requested to join the i^th^ fog core, but are denied due to the fullness of the particular fog core. The average number of denied packet is derived as

(25) $$\lambda_{m}P_{M}^{i}$$

The effective packet is obtained as

(26) $$\lambda_{e}=\lambda_{m}\left(1-P_{M}^{i}\right)$$

The throughput of each fog core is the total amount of packets served during the defined period of time expressed as

(27) $${{\bar{T}}_{m}}^{i}=\lambda_{m}\left(1-P_{B}^{i}\right)$$

The utilization of each fog core is expressed as

(28) $${\upsilon^{i}}_{m}=\frac{{{\bar{T}}_{m}}^{i}}{s\mu_{m}}=\frac{\rho_{s}}{s}\left(1-P_{B}^{i}\right)$$

The mean number of packet requests for each fog core is

(29) $${{\bar{M}}_{m}}^{i}=\sum_{a=1}^{M}\;aP_{a}^{i}$$

The mean number of packet request for each fog core and waiting in queue is expressed as follows:

(30) $${{\bar{Q}}_{m}}^{i}=\sum_{a=s+1}^{M}\;(a-s)P_{a}^{i}$$

Lastly, using Little’s law, the mean response time and the mean waiting time for each fog core are obtained as follows:

(31) $$\bar{E{{\left[R\right]}_{m}}^{i}}=\frac{{{\bar{M}}_{m}}^{i}}{\lambda_{m}\left(1-P_{B}^{i}\right)}$$

(32) $${\bar{E\left[w\right]}}_{m}^{i}=\frac{{{\bar{Q}}_{m}}^{i}}{\lambda_{m}\left(1-P_{B}^{i}\right)}$$

We obtain the performance equations for the fog subsystem after obtaining the main performance metrics equation for the single fog core. We begin with the blocking probability for fog cores in the subsystem:

(33) $$P_{M}=\sum_{i=1}^{H}\;P_{M}^{i}$$

The mean number of packet requests in overall fog cores is obtained as

(34) $${\bar{M}}_{m}=\sum_{i=1}^{H}\;{{\bar{M}}_{m}}^{i}$$

The mean number of packets waiting in the fog core’s queue is obtained as follows:

(35) $${\bar{Q}}_{m}=\sum_{i=1}^{H}\;{{\bar{Q}}_{m}}^{i}$$

The mean throughput of the fog subsystem is obtained as follows:

(36) $${\bar{T}}_{m}=\sum_{i=1}^{H}\;{{\bar{T}}_{m}}^{i}$$

Finally, the mean response time and the mean waiting time for the fog subsystem are obtained as

(37) $$\bar{E{\left[R\right]}_{m}}=\frac{{\bar{M}}_{m}}{{\bar{T}}_{m}}$$

(38) $$\bar{E{\left[w\right]}_{m}}=\frac{{\bar{Q}}_{m}}{{\bar{T}}_{m}}$$

### 5.3. Other Recommendations

This section describes each virtual CPU core in the CCDC subsystem. Each vCPU follows the M/M/t/C queueing model. The packets processed in the fog either leave the system with a probability of $q_{m}$ or are submitted to the CCDC subsystem with a probability of $P_{md}$. Table 4 lists each parameter used in this section. In the M/M/t/C queueing system, the number of vCPU core is “t”. Each CPU core can accept and process “C” packets.

The process of packet arrival and departure follows the birth–death process with an arrival rate a and steady-state equations expressed as follows:

$P_{C}$ = probability c packets in the system

(39) $$P_{C}\left\{\begin{array}{l} \frac{P}{C!}{\left(\frac{\lambda_{d}}{\mu_{d}}\right)}^{C}P_{0},\;\;\;for\;1\leq C<t\\ \frac{P({\lambda_{d}}^{c})}{t!t^{C-t}\mu_{d}^{C}}P_{0},\;\;\;for\;C\geq t \end{array}\right.$$

where *ρ* indicates the utilization of a single server

(40) $$\rho_{d}=\frac{\lambda_{d}}{\mu_{d}}$$

The utilization of *t* servers in the system is expressed as

(41) $$\rho_{t}=\frac{\lambda_{d}}{t\mu_{d}}$$

$P_{o}$ indicates the probability when the system is empty. We obtain $P_{o}$ by normalization:

(42) $$P_{0}={\left[1+\sum_{i=1}^{t-1}\;\frac{{\left(t\rho \right)}^{i}}{i!}+\frac{{\left(t\rho \right)}^{t}\left(1-\rho^{C+1-t}\right)}{t!\left(1-\rho \right)}\right]}^{-1}$$

The main performance metrics are obtained in this part. First, the blocked probability is acquired:

(43) $$P_{B}^{i}=P_{C}^{i}$$

The term $P_{B}^{i}$ represents the probability of packets requesting to join to the system but being denied due to the fullness of system queue and blocked. The average number of denied packet is derived as

(44) $$\lambda_{d}P_{C}^{i}$$

The effective packet is obtained as

(45) $$\lambda_{e}=P_{md}\lambda_{d}\left(1-P_{C}^{i}\right)$$

The throughput of each vCPU core is the total amount of packets served during the defined period of time expressed as follows:

(46) $${{\bar{T}}_{d}}^{i}=P_{md}\lambda_{d}\left(1-P_{B}^{i}\right)$$

The utilization of each vCPU core is expressed as

(47) $${\upsilon^{i}}_{d}=\frac{{{\bar{T}}_{d}}^{i}}{d\mu_{d}}=\frac{\rho_{t}}{t}\left(1-P_{B}^{i}\right)$$

The mean number of packet request to join each vCPU core is obtained as

(48) $${{\bar{M}}_{d}}^{i}=\sum_{a=1}^{C}\;aP_{a}^{i}$$

The mean number of packet request for each vCPU core and waiting in queue is expressed as follows:

(49) $${{\bar{Q}}_{d}}^{i}=\sum_{a=t+1}^{c}\;(a-t)P_{a}^{i}$$

Finally, the mean response time and the mean waiting time are obtained using Little’s formula:

(50) $$\bar{E{{\left[R\right]}_{d}}^{i}}=\frac{{{\bar{M}}_{d}}^{i}}{\lambda_{d}\left(1-P_{B}^{i}\right)}$$

(51) $${\bar{E\left[w\right]}}_{d}^{i}=\frac{{{\bar{Q}}_{d}}^{i}}{\lambda_{d}\left(1-P_{B}^{i}\right)}$$

Finally, we obtain the performance equations for the CCDC subsystem after obtaining the main performance equation for the single vCPU core. The total blocking probability for the CCDC cores in the subsystem is written as follows:

(52) $$P_{C}=\sum_{i=1}^{X}\;P_{C}^{i}$$

The mean number of packet requests to the overall CCDC core is expressed as shown:

(53) $${\bar{M}}_{d}=\sum_{i=1}^{X}\;{{\bar{M}}_{d}}^{i}$$

The mean number of packets waiting in the CCDC core’s queue is expressed as follows:

(54) $${\bar{Q}}_{d}=\sum_{i=1}^{X}\;{{\bar{Q}}_{d}}^{i}$$

The mean throughput of the CCDC subsystem is obtained as

(55) $${\bar{T}}_{d}=\sum_{i=1}^{X}\;{{\bar{T}}_{d}}^{i}$$

Lastly, the mean waiting time and the mean response time for the CCDC subsystem are given as

(56) $$\bar{E{\left[R\right]}_{d}}=\frac{{\bar{M}}_{d}}{{\bar{T}}_{d}}$$

(57) $$\bar{E{\left[w\right]}_{d}}=\frac{{\bar{Q}}_{d}}{{\bar{T}}_{d}}$$

### 5.4. Overall System Performance

In this section, we calculate the performance of the slice where Cloud-RAN, fog, and CCDC are used. First, the total throughput of the H-IoT slice in the proposed queueing model equal to the throughput of each subsystem (Cloud-RAN, Fog, and CCDC) is presented as

(58) $$\bar{T}={\bar{T}}_{c}+{\bar{T}}_{m}+{\bar{T}}_{d}$$

The mean response of each H-IoT slice in the proposed queueing model is the total of the mean response time for the three subsystems:

(59) $$\bar{E\left[R\right]}=\bar{E{\left[R\right]}_{c}}+\bar{E{\left[R\right]}_{m}}+\bar{E{\left[R\right]}_{d}}$$

Next, the mean waiting time for each H-IoT slice in the proposed queueing model is the total mean waiting time for each subsystem:

(60) $$\bar{E\left[w\right]}=\bar{E{\left[w\right]}_{c}}+\bar{E{\left[w\right]}_{m}}+\bar{E{\left[w\right]}_{d}}$$

The mean number of packet requests for each H-IoT slice is the total number of packet requests for each subsystem:

(61) $$\bar{M}={\bar{M}}_{c}+{\bar{M}}_{m}+{\bar{M}}_{d}$$

The mean number of packets waiting in the queue for each H-IoT slice is the total number of packets waiting in the queue for each subsystem:

(62) $$\bar{Q}={\bar{Q}}_{c}+{\bar{Q}}_{m}+{\bar{Q}}_{d}$$

The blocking probability of the H-IoT slice is the number of requests denied in each subsystem obtained as

(63) $$P_{block}=P_{K}+P_{M}+P_{C}$$

Lastly, the system cost can be obtained as

(64) $$Cost_{s}=C_{vBBU}.S_{vBBU}+C_{vfog}.S_{vfog}+C_{vCPU}.S_{vCPU}$$

where $C_{vBBU}$ is the total number of cores in the Cloud-RAN subsystem; $C_{vfog}$ is the total number of virtual servers in the fog subsystem; $C_{vCPU}$ indicates the total number of vCPU in the CCDC subsystem; $S_{vBBU}$ indicates the service cost for each virtual BUU core in the Cloud-RAN; $S_{vfog}$ indicates the service cost for each vServer in the fog subsystem; and $S_{vCPU}$ indicates the service cost for each vCPU in the CCDC subsystem.

The cost of the expected waiting time of packet requests in the slice is obtained as follows:

(65) $$Cost_{w}=\lambda \bar{E\left[w\right]}C_{w}$$

where the total arrival rate for packets into the slice is λ, and the mean waiting time of the packets in the slice is $\bar{E\left[w\right]}$. The cost of waiting time for the packet requests refers to $C_{w}$. Thus, the overall cost of the slice is calculated as follows:

(66) $$Cost=Cost_{s}+Cost_{w}$$

### 5.5. Experimental Results

#### 5.5.1. Simulation Parameters

This section introduces a numerical example to validate the analytical model proposed in the previous section. Therefore, the results in this section are obtained by using two different methods based on: (1) the performance measure equations from the queueing model in Section 5; and (2) the JSIMgraph tool from the JMT simulation tools. JMT is an open-source software comprising six different tools for performance evaluation, capacity planning, workload characterization, and modeling computer and communication systems. The JMT provides a discrete-event simulator with two different interfaces for analyzing queueing networks: alphanumerical (JSIMwiz) and graphical (JSIMgraph) [26].

The different performance indices are analyzed and calculated using a single network slice. The other two slices are calculated in the same manner. Table 5 shows the main parameters used in this example.

#### 5.5.2. Results and Discussion

As demonstrated by the blotted figures in this section, the results validated the proposed queueing model and verified that it can be used to enhance the main performance measures. Three different scenarios were applied to measure the effect of the virtual BBU cores on the system performance, the number of fog cores on system delays, and the virtual BBU and fog cores on the overall system cost. The plotted curves for the previous analytical equations and the JMT simulation produced similar results. This should verify the validation and efficiency of the proposed analytical model. The plotted graphs in this section measured the main performance parameters.

**Experiment** **1.** *Effect of Virtual BBU Nodes on The System Performance.*

In this experiment, we measured the effect of virtual BBU cores on the key performance indices of the system: CPU utilization Equation (9), throughput Equation (58), response time Equation (59), waiting time Equation (60), number of requests in the system Equation (61), and system loss Equation (63). We varied the arrival rate from 100,000 packets/s to 1,000,000 packets/s to evaluate the response of the virtual BBU cores with an increase in load. The number of virtual BBU cores was 26, 32, 38, 44 cores.

**Figure 3:**
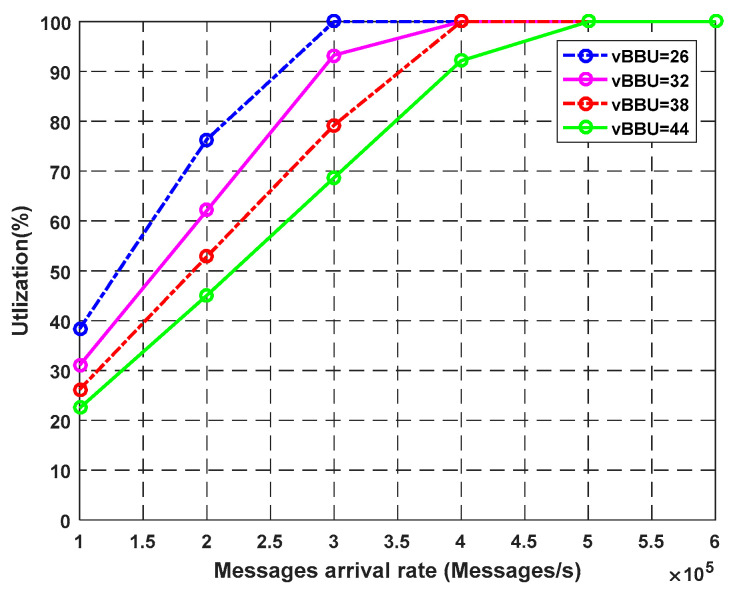
Impact of vBBU cores on CPU utilization.

Figure 3 shows the effects of the virtual BBU cores on the CPU utilization. The amount of work handled by a CPU increases as the load or arrival rate increases. Using more virtual BBU cores, the system has more opportunities to process larger requests using inactive cores, leading to a minimum rate of CPU utilization. The graph shows that when the system arrival rate is equal to 300k packets/s, different rates are observed for the CPU utilization based on the number of virtual BBU cores. By using fewer cores (e.g., 26 cores), the processing units reach their maximum capacity, and the CPU has 100% usage. At this point, it will remain fixed over time, and the other performance measures will definitely deteriorate, as illustrated in previous analytical equations.

**Figure 4:**
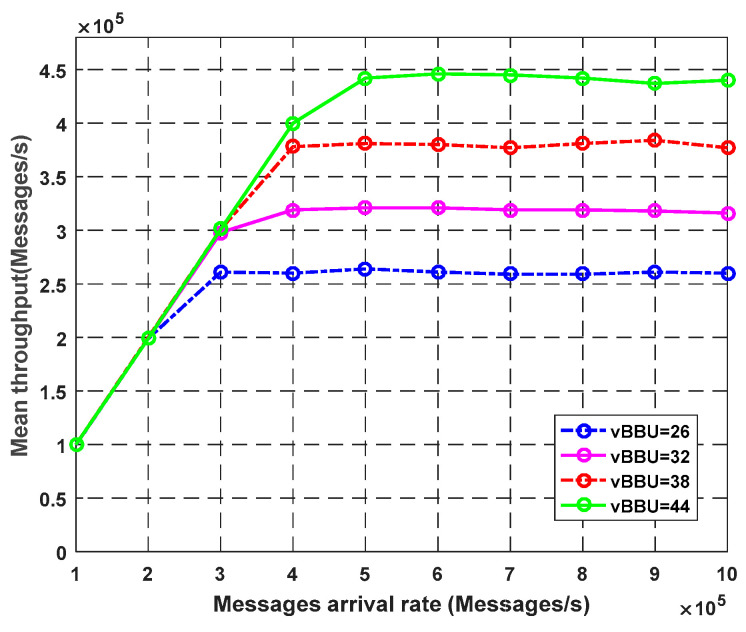
Impact of vBBU cores on throughput.

Figure 4 illustrates the effect of the virtual BBU cores on the mean response time of the system. The response time refers to the total amount of time the system takes to respond to a request, including processing and transmission time. Using fewer processing units makes those that remain overload at a faster rate and leads to a bottleneck. As proven by the graph, using more cores decreases the response time of the requests. Furthermore, using 26 cores with an arrival rate of 300k packets/s results in a 100% rate for the CPU usage (Figure 3), which leads to an increase in the response time from 0.22 to 1.28 ms. For eHealth applications, most of the services are time-sensitive and need to minimize the response time as much as possible.

**Figure 5:**
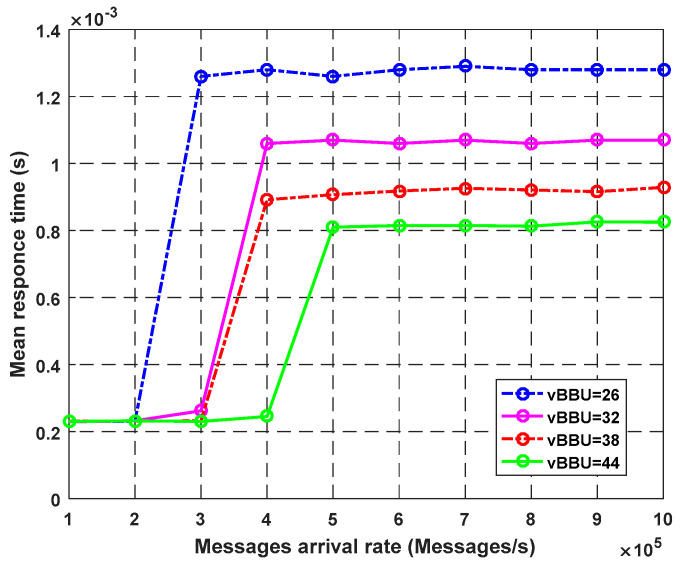
Impact of vBBU cores on average response time.

Figure 5 demonstrates the impact of virtual BBU cores on the other key performance parameters. It shows the observed throughput with different arrival rates. The throughput increases in proportion to an increase in the arrival rate. Increasing the number of virtual BBU cores improves the system throughput or the number of requests fulfilled per second. At the arrival rate of 700k packets/s, using 44 cores instead of 26 increased the mean throughput by more than 180,000 packets/s.

**Figure 6:**
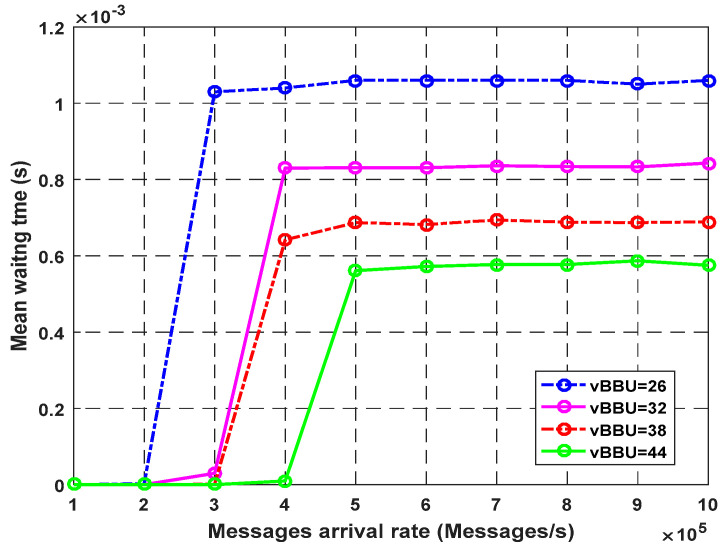
Impact of vBBU cores on average waiting time.

Figure 6 describes the average waiting time of the system as a function of the arrival rate for different virtual BBU cores. The average waiting time continues to increase with the arrival rate. As seen in 500k packets/s, using more cores decreases the waiting time of messages in the queue. However, after some arrival rate values, the mean waiting time remained almost unchanged because the CPU utilization approached its maximum capacity, and no inactive processing units can be used.

**Figure 7:**
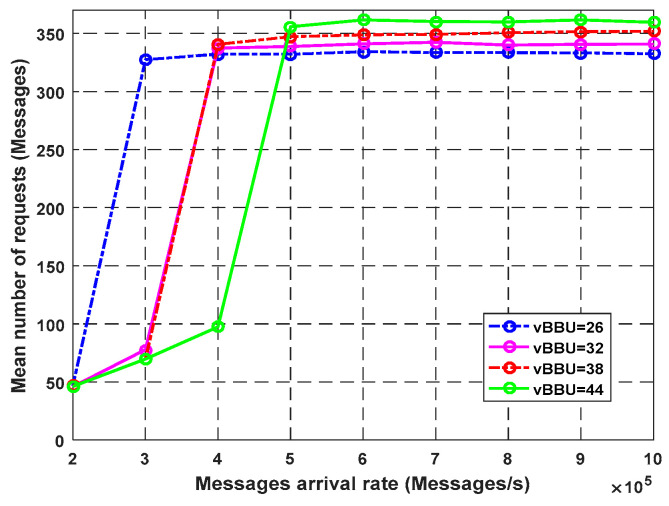
Impact of vBBU cores on average number of requests.

**Figure 8:**
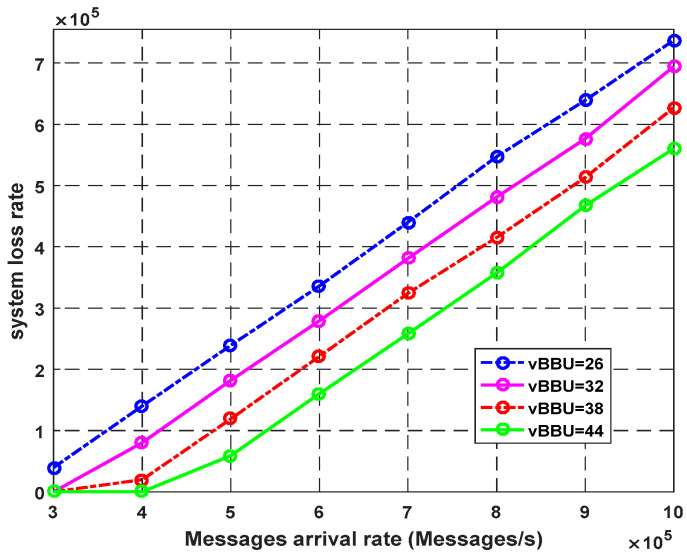
Impact of vBBU cores on system loss rate.

The average number of requests in the system increased with an increase in the arrival rate (Figure 7). The increases in the cores of the virtual BBU improved the system and made the processing units operate at a higher number of requests. For example, at the arrival rate of 600k packets/s, the average number of messages for 44 virtual BBU cores was higher than that using 26 cores. When the CPU was fully utilized, the virtual BBU cores became busy serving the requests. The queue size filled up. The throughput was saturated. Moreover, the average number of packet requests, average response time, and average waiting time unpredictably grew and remained constant. At that point, the drop rate increased (Figure 8).

Figure 8 shows the effect of the number of virtual BUU cores on the system loss rate. Increasing the number of virtual BBU cores led to a decrease in the number of rejected requests. For example, at the arrival rate of 1000k packets/s, with 32 cores, the system loss rate indicated 694k packets/s. With 44 cores, the system loss rate indicated 560k packets/s. Hence, increasing the number of vBUU cores improved the system performance and the QoS requirements for eHealth services.

**Experiment** **2.** *Effect of Fog Nodes on The System Response Time.*

**Figure 9:**
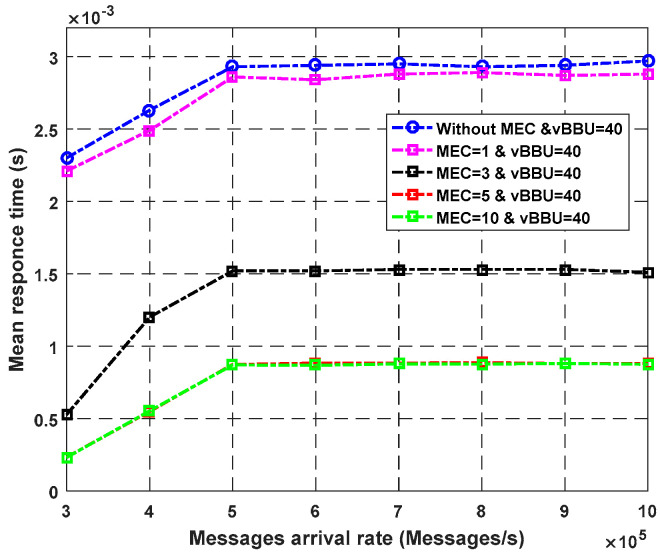
Impact of MEC nodes on average response time.

**Figure 10:**
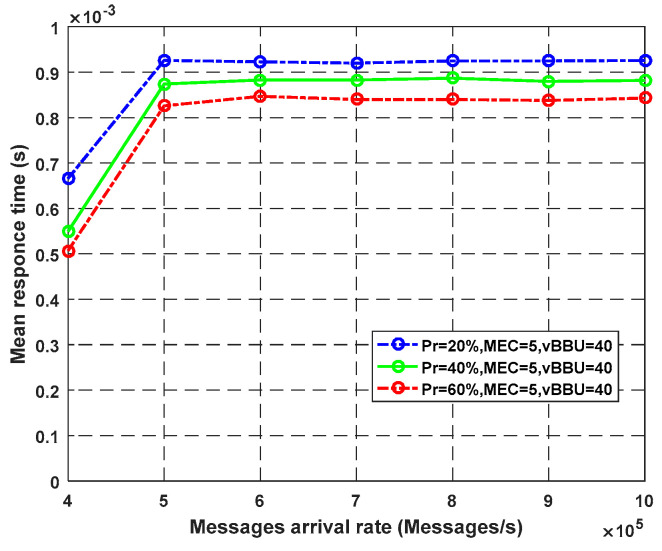
Impact of MEC nodes on average response time according to probability P_ec_.

The plotted graphs in this experiment show the effect of varying the arrival rate of packets the fog nodes on the system response time Equation (56). Figure 9 depicts the observed response time with and without fog cores. The number of virtual BBU cores was fixed and set to be equal to 40. The number of fog core nodes used to handle critical requests was 1, 3, 5, and 10; alternatively, no core was used. As noticed from the graph, the highest response time was achieved using the system without fog cores. When using more fog cores, the response time decreased, and the system performance increased. However, after crossing a limited value of the fog cores, no change was observed in the mean response time, as seen with the five- and 10-core curves. Figure 10 illustrates the impact of using fog cores with the ability to complete the execution at the fog servers (q). A higher number of (q) shows a significant improvement in terms of the response time because the service is complete at the edge instead of the cloud servers. For eHealth services, we need to insert fog cores to satisfy the low latency and QoS requirements of sensitive sensed data.

**Experiment** **3.** *Effect of Virtual BBU and Fog Cores on the Overall System Cost.*

**Figure 11:**
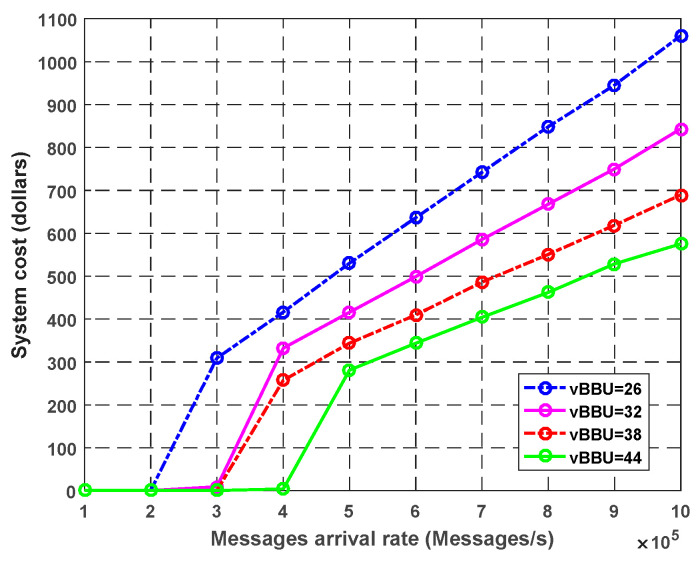
Effect of the number of virtual BBU cores on the system cost.

In this experiment, we show the effect of the number of virtual BUU and fog cores on the overall system cost with an increase in the arrival rate. According to Equation (66), the total system cost was calculated by adding the costs of the waiting time and the service for each system core. Figure 11 illustrates the effect of the virtual BBU cores on the system cost. We assumed that the waiting time cost was 1$ for every second. The service cost for each virtual BBU core was equal to 0.01$ for every second. We wanted to focus on the effect of the virtual BBU core; thus, we neglected the cost for the other subsystem fog and CCDC.

In Figure 11, we assumed that the fog and CCDC costs were constant. We noticed that as the packet arrival rate increased, the system cost (in dollars) increased withal. When we increased the number of virtual BUU cores, the total system cost was decreased. For example, at the arrival rate of 1000k packets/s, the system cost with 44 cores was 590$; conversely, the with 26 cores increased to 1050$. Consequently, using more virtual BBU cores decreased the cost of the total system and enhanced the QoS requirements.

**Figure 12:**
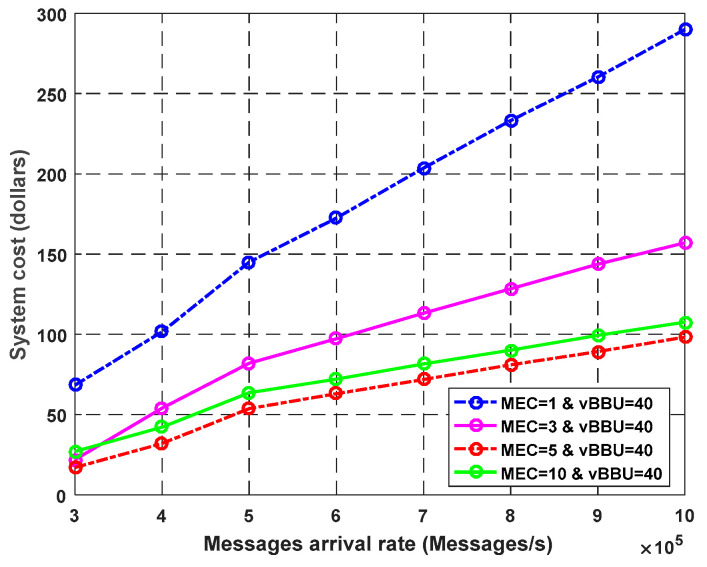
Effect of the number of fog cores on the system cost.

Figure 12 illustrates the effect of the fog cores on the total system cost. The results were obtained by assuming that the waiting time cost was equal to 1$/s, and the service cost for each fog core was equal to 2$/s. We used a constant value for the Cloud-RAN and CCDC costs. The curves show a significant decrease in the total system cost as the number of fog cores increased. After proceeding with a certain value for a number of fog cores, the fog cores became unusable, and there was waiting time, leading to an increase in the total cost as the number of cores increased. Consequently, increasing the fog cores (limited by a threshold value) decreased the total cost and improved the system performance.

The previous figures and results showed how the system can be used to estimate the number of computing resources required in each subsystem and how this model can be used to enhance the overall QoS requirements of different eHealth service applications.

## 6. Conclusions

This study shows an architecture for eHealth IoT systems using 5G network slicing. The proposed paradigm was composed of Cloud-RAN, fog, and CCDC for each eHealth slice. Using the queue model, we derived the formula of performance metrics measurements, such as the throughput, CPU utilization, system blocking rate, mean number of packet requests, mean number of packet requests waiting in the queue, mean response time, and mean waiting time. We also presented herein a numerical example to validate the queueing model and show how the system is used to estimate the number of computing resources required. The results of the JMT simulation and the analytical equations were close to each other. The overall QoS requirements of eHealth services can be enhanced by increasing the number of virtual BBU and fog nodes. In future work, AI will be used to optimize the number of resources needed to achieve the system QoS in different environments.

## Figures and Tables

**Table 1:** Classifications of eHealth services and their QoS requirements.

eHealth Classifications	QoS Requirements	Example
Online consultation	High bandwidth Video caching Low latency in emergencies	Teleconsultation between patient/doctor or doctor/doctor using biological sensor data
Remote monitoring	Security High reliability Long battery life Small data volume	Telemonitoring the vital signs of cardiovascular patients
Remote surgery	<300 ms RTT [4] High reliability	Robotic surgery

**Table 2:** Queueing parameters of Cloud-RAN.

Parameters	Description
$\lambda_{c}$	Packet arrival rate to Cloud-RAN subsystem
${1/\mu }_{c}$	Average service time of Cloud-RAN
$P_{k}$	Probability of K packets in the system
$\rho_{c}$	Utilization of each virtual BBU core
b	Number of virtual BBU in each core
K	Maximum number of accepted packets in each virtual BBU core
F	Total number of virtual BBU cores in the Cloud-RAN subsystem
$P_{B}^{i}$	Blocking probability in the *i*^th^ slice
$\lambda_{e}$	Effective rate of arrival message
${{\bar{T}}_{c}}^{i}$	Throughput service for single virtual BBU core
${\upsilon^{i}}_{c}$	Utilization service for single virtual BBU core for each virtual BBU core
${{\bar{Q}}_{c}}^{i}$	Mean number of packets requests waiting in queue for each virtual BBU
$\bar{E{{\left[R\right]}_{c}}^{i}}$	Mean response time for each virtual BBU core
$\bar{E{{\left[R\right]}_{c}}^{i}}$	Mean waiting time for each virtual BBU core in Cloud-RAN
${\bar{M}}_{c}$	Mean number of packets requests in Cloud-RAN
${\bar{Q}}_{c}$	Mean number of packets waiting in Cloud-RAN queue
${\bar{T}}_{c}$	Mean throughput of overall Cloud-RAN
$\bar{E{\left[R\right]}_{c}}$	Mean response time in Cloud-RAN
$\bar{E{\left[w\right]}_{c}}$	Mean witting time in Cloud-RAN

**Table 3:** Queueing parameters of the Fog Subsystem.

Parameters	Description
$\lambda_{m}$	Arrival rate of packets to fog
1/$\mu_{m}$	Mean service time of vServers in fog
$P_{m}$	Probability of *m* packets in a single fog core
$\rho_{m}$	Utilization parameter of a single fog core
s	Number of virtual servers in each fog core
M	Maximum number of accepted packets in each fog core
H	Total number of fog cores in fog subsystem
$P_{c}^{i}$	Blocking probability in each fog core
${{\bar{T}}_{m}}^{i}$	Throughput of a fog core
${\upsilon^{i}}_{m}$	Utilization of each fog core
${{\bar{M}}_{m}}^{i}$	Mean number of packet requests for each i^th^ fog core
${{\bar{Q}}_{m}}^{i}$	Mean number of packet requests waiting in i^th^ fog core queue
$\bar{E{{\left[R\right]}_{m}}^{i}}$	Mean response time for each fog core
$\bar{E{{\left[W\right]}_{m}}^{i}}$	Mean waiting time for each fog core
${\bar{M}}_{m}$	Mean number of packet requests
${\bar{Q}}_{m}$	Mean number of packet requests waiting in the fog subsystem
${\bar{T}}_{m}$	Mean throughput of the fog subsystem
$\bar{E{\left[R\right]}_{m}}$	Mean response time in the fog subsystem
$\bar{E{\left[w\right]}_{m}}$	Mean waiting time in fog subsystem

**Table 4:** Queueing parameters of the CCDC subsystem.

Parameters	Description
$P_{md}\lambda_{d}$	Arrival rate of packets into the CCDC subsystem
1/$\mu_{d}$	Mean service time of vCPU in the CCDC subsystem
$P_{C}$	Probability of C packet in each vCPU
$\rho_{t}$	Utilization parameter of a single vCPU
t	Number of virtual CPU in each CCDC core
C	Maximum number of packets in each CCDC core
x	Total number of CPU core in the CCDC subsystem
$q_{m}$	Probability of packets leaving the system from fog
$P_{md}$	Probability of packet transfer from fog to CCDC
$P_{d}^{i}$	Blocking probability at vCPU
${{\bar{T}}_{d}}^{i}$	Throughput for each CPU core
${\upsilon^{i}}_{d}$	Utilization of each CPU core
${{\bar{M}}_{d}}^{i}$	Mean number of packet requests for each CPU core
${{\bar{Q}}_{d}}^{i}$	Mean number of packet request waiting in queue for each CPU core
$\bar{E{{\left[R\right]}_{d}}^{i}}$	Mean response time for each CPU core
$\bar{E{{\left[W\right]}_{d}}^{i}}$	Mean waiting time for each CPU core
${\bar{M}}_{d}$	Mean number of packet requests in the CCDC
${\bar{Q}}_{d}$	Mean number of packet requests waiting in the CCDC queue subsystem
${\bar{T}}_{d}$	Mean throughput of the CCDC subsystem
$E{\left[R\right]}_{d}$	Mean response time in the CCDC subsystem
$\bar{E{\left[w\right]}_{d}}$	Mean waiting time in the CCDC subsystem

**Table 5:** Queueing parameters of the JMT simulation.

E	Description	Value
*λ*	Packet arrival rate	[100,000 to 1,000,000] (Packets/s)
1/$\mu_{c}$	Mean service time of Cloud-RAN	0.0001 (s)
1/$\mu_{m}$	Mean service time of vServers in fog	0.00001 (s)
1/$\mu_{d}$	Mean service time of vCPU in the CCDC subsystem	0.0002 (s)
K	Maximum number of packets in each virtual BBU core	300
H	Total number of fog cores in the fog subsystem	5
M	Maximum number of packets in each fog core	200
$q_{m}$	Probability of packets leaving system from the fog	0.4
x	Total number of CPU cores in the CCDC subsystem	20
t	Number of vCPU in each CCDC core	10
C	Maximum number of packets in each CCDC core	500

## Data Availability

The data that support the findings of this study are available from the corresponding author upon reasonable request.
